# Sleep quality as a mediator between family function and life satisfaction among Chinese older adults in nursing home

**DOI:** 10.1186/s12877-024-04996-1

**Published:** 2024-04-29

**Authors:** Wenfen Zhu, Yutong Wang, Jiao Tang, Fangyi Wang

**Affiliations:** School of Nursing, Chongqing Medical University, #1 Yixueyuan Road, Yuzhong District, Chongqing, China

**Keywords:** Sleep quality, Life satisfaction, Family function, Nursing home, Older adults

## Abstract

**Background:**

The life satisfaction of the elderly in nursing home is the focus of social concern.The purpose of this study was to evaluate the effects of family function and sleep quality on life satisfaction among elderly individuals in nursing homes and examine the mediating effect of sleep quality between family function and life satisfaction.

**Methods:**

A cross-sectional observational study was conducted .A total of 127 older adults who completed the Life Satisfaction Index A (LSI-A), the Family APGAR Index and the Pittsburgh Sleep Quality Index (PSQI) were recruited from four nursing homes in Chongqing, China.

**Results:**

Life satisfaction was positively correlated with family function (*r*=0.434, *p*<0.01) and negatively correlated with PSQI (*r* = -0.514, *p*<0.01). PSQI was found to be negatively associated with family function (*r*=-0.387, *p*<0.01).Family function had a significant effect on PSQI (path a: β=-0.8459, 95% CI=-1.2029, -0.4889), and PSQI had a significant effect on life satisfaction (path b: β=-0.3916, 95% CI=-0.5407, -0.2425). The total effect (path c) and direct effect (path c') of family function on life satisfaction were significant (β=0.8931, 95% CI=0.5626, 1.2235 and β=0.56181, 95% CI=0.2358, 0.8879, respectively). The coefficient for the indirect effect of family function on life satisfaction through PSQI was statistically significant (β=0.3312, 95% CI=0.1628, 0.5588). PSQI played a partial mediating role between family function and life satisfaction, and PSQI mediated 32.58% of the total effect of family function on life satisfaction.

**Conclusions:**

Family function and sleep quality were significant predictors of elderly people's life satisfaction in nursing homes. Sleep quality partially mediated the relationship between family function and life satisfaction.The interventions focused on promoting family function and improving sleep quality may be more helpful in improving elderly people's life satisfaction in nursing homes.

## Introduction

Population aging has become a worldwide concern, including in China. According to the results of China's seventh census, the number of elderly individuals aged 60 or above reached 264 million in 2020, accounting for 18.7% of China's total population, and the proportion is expected to reach approximately 25% by 2030 [1]. "Pension difficulty" has become a prominent concern in China [2]. Promoting the life satisfaction and health status of older people was a major task for Chinese policy-makers, researchers and health care providers.^3^ Due to the change in family size and population migration caused by economic development in China, the traditional family pension model was increasingly unable to meet the needs of the elderly [3]. Older adults living in nursing homes (NHs) are becoming more common in China, although they prefer to live in their own homes^4^,Elderly people in NHs were an important part of the elderly population [4]. So, Promoting the health and life satisfaction of older adults in NHs is,a major public concern.

Life satisfaction is subjective measure of wellbeing and an important measure for assessing physical and mental health [5, 6] and quality of life [7]. Assessing people's life satisfaction can help to distinguish the level of quality of life and thus guide interventions to enhance their quality of life [8]. Life satisfaction was also an important consideration of health management for older adults [9]. The life satisfaction of elderly individuals in NHs was lower than that of elderly individuals in homes [10]. Previous studies have shown that sociodemographic characteristics, health status and relationships among family members may influence the life satisfaction of elderly individuals [11, 12]. Older adults in NHs have challenging deteriorated physiological functions with age, and living in NHs could lead to a decrease in family communication.Therefore, it is important for professionals in the field of elderly care to evaluate the influencing factors of life satisfaction and develop interventions to improve their life satisfaction.

Family functioning refers to interactions with family members that involve physical,emotional,and psychological activities and affect many aspects of family life [13]. Good family functioning can provide good conditions to support the development of family members. Previous studies have shown that family function has a positive correlation with the life satisfaction of elderly persons [14]. Families were the primary source of emotional and social support [15]. Most older adults in China hope to live at home [16]. However, with ongoing changes to Chinese society, the traditional family pension model for the aged was gradually weakened, and the elderly had to choose NHs for the aged [16]. This may lead to increased emotional demands for the elderly in NHs. Studies have shown that the emotional needs of older adults in NHs were greater than those of community-dwelling older adults [17]. At present in China, many staffs of NHs often ignored the psychological needs of the elderly and only focused on meeting their basic life needs [18]. The level of family support was lower for the elderly in NHs, which could not meet the needs of the elderly for family and affection [19]. Older adults in NHs may face more stress when integrating into the new living environment, and their emotional demands on their family may affect their life satisfaction. However, little is known about the level of family function among NH residents, as well as the impact of their family function on life satisfaction.

Sleep quality is defined as “an individual’s self-satisfaction with all aspects of the sleep experience” [20]. Good sleep quality would lead to a successful older adult's life,and was associated with high life satisfaction [21]. Sleep could affect the ability of people to regulate their emotions [22] and influence physical and mental health [23]. Previous studies have found that sleep quality have strong correlation with life satisfaction in older adults and were mediated by health status [24]. Sleep quality was affected by environment [25], lifestyle and health status [26]. Older adults in NHs are under pressure to adapt to new lifestyles and environments,this may lead to decreased sleep quality . Previous studies have suggested that elderly people in NHs have poor sleep quality, and the level of sleep quality was lower than that of elderly people living at home [27, 28]. Sleep quality may be important to improve life satisfaction for older adults in NHs .However, a limited number of studies have examined elderly people's sleep quality and the relationship between sleep quality and life satisfaction in NHs. Studies showed that sleep quality was associated with family function [29], good family function contributed to the improvement of sleep quality and family function directly predicted sleep quality [30]. Previous studies showed that poor family relationships were significantly associated with poor sleep quality [31], and negative family interactions were linked with poor sleep quality among older adults [32]. Family support was positively correlated with good sleep quality in the elderly [33], and the olders who experienced family members' abuse were more likely to report poor sleep quality [34]. The older adults had more complete family functions and got more family support, corresponding to better sleep quality [35]. Literature has also shown that sleep quality has a mediating effect on family function and quality of life among parents of children with home mechanical ventilation [36]. Life satisfaction was used as an important indicator to measure the quality of life [7], so sleep quality may be a mediating factor between family function and life satisfaction among olders in NHs.

Based on the above, the aim of this study was to examine the relationship between family function, life satisfaction and sleep quality among NHs elderly individuals. Our hypotheses for the elderly in nursing homes were as follows: (1) family functioning has a positive effect on life satisfaction and good sleep quality; (2) sleep quality plays a mediating role between family functioning and life satisfaction.

## Methods

### Design and participants

This study had a cross-sectional design. Adopting the method of convenience sampling, 127 older adults were recruited from four nursing homes (≥100 beds) in Chongqing city, China. The inclusion criteria were as follows: (1) staying in a nursing home for more than 6 months; (2) aged 60 years and above; (3) no serious mental or physical disease; (4) no cognitive impairment or impaired consciousness; (5) able to take care of themselves; (6) voluntarily participating in this survey.

G*Power3.1.9.7 software was used to estimate the required sample size. Calculations showed that 123 participants would be required for statistical power (1-β err prob= 0.90), effect size (f^2^= 0.15), significance level (α err prob=0.05), and 6 predictors [37]. According to the criteria proposed by Matthew S. Fritz and David P. MacKinnon for the sample size required to detect mediating effects [38], with path a=0.26, path b=0.39 and power=0.80, a bias-corrected bootstrapping procedure requires a minimum sample population of 118 people.

### Data collection

Data were collected by well-trained research assistants from January to July 2023. With the help of staff from four nursing homes,we visited 154 eligible participants, 140 participants agreed to participate in the survey.

A face-to-face interview approach was adopted to collect data using a self-report questionnaire. For the elderly who were illiterate or had poor vision, the investigator read out the questions of the questionnaire in turn, and after the elderly dictated them, the questionnaires were filled out by the investigators on their behalf. Before the data collection, all participants were informed of the objective of the survey and promised them to keep their information confidential. Each interview took approximately 20-25 minutes. All study procedures were approved by the Ethics Committee of Chongqing a University.

### Instruments

Study instruments included three scales measuring family function, life satisfaction and sleep quality, as well as social demographic characteristics.

Social demographic characteristics included age, gender, education level, marital status, family visitation and monthly pension. These were classified as follows: age(60-69,70-79,80-89 and ≧90 years), gender(female, male), education level(≦primary school, middle school, high school, and≧ college), marital status(married, unmarried or divorced or others), and monthly pension (<2000RMB, 2000-4000RMB, and >4000RMB).

Family function was measured by the Family APGAR Index. APGAR was developed by Smilkstein in 1978. It consists of five items and includes five domains: adaptability (A), partnership (P), growth (G), affection (A), and resolve (R) [39]. A 3-point Likert scale from 0 (hardly ever) to 2 (almost always) was used for each item. The total score ranged from 0 to 10. Higher APGAR scores indicate better family functioning [40, 41]. The Chinese version of the family APGAR index has been widely used with satisfactory validity and reliability [42]. The Cronbach’s α of the APGAR was 0.728 in this study.

Sleep quality was measured using the Pittsburgh Sleep Quality Index (PSQI) [43]. The PSQI contains seven dimensions: subjective sleep quality, sleep latency, sleep duration, habitual sleep efficiency, sleep disturbance, use of sleeping medication, and daytime dysfunction. The total score of the seven dimensions ranged from 0 to 21 points. A higher score represents poorer sleep quality. The PSQI was used to evaluate the sleep conditions of individuals during the past month. The Chinese version of the PSQI is a reliable and valid assessment tool for sleep quality [44, 45]. In this study, the Cronbach’s α of the PSQI was 0.718.

Life satisfaction was measured using the Life Satisfaction Index A (LSI-A) [46]. The LSI-A consists of 20 items. Participants filled in ‘agree’, ‘disagree’ or ‘undecided’ with each item. Each item was scored 0 or 1 (for 12 items, ‘agree’ = 1; for 8 items, ‘disagree’ = 1), and the total scores of LSI-A ranged from 0 to 20. The higher the score is, the higher the level of life satisfaction. The LSI-A had good reliability and validity [46, 47]. The Cronbach’s α was 0.713 in this study.

### Ethical considerations

The study protocol was approved by the ethic committee of Chongqing Medical University. All participants were informed about the right to decline answer any questions or withdraw from the survey.The survey was voluntary and anonymous.

### Statistical analysis

**Fig. 1:**
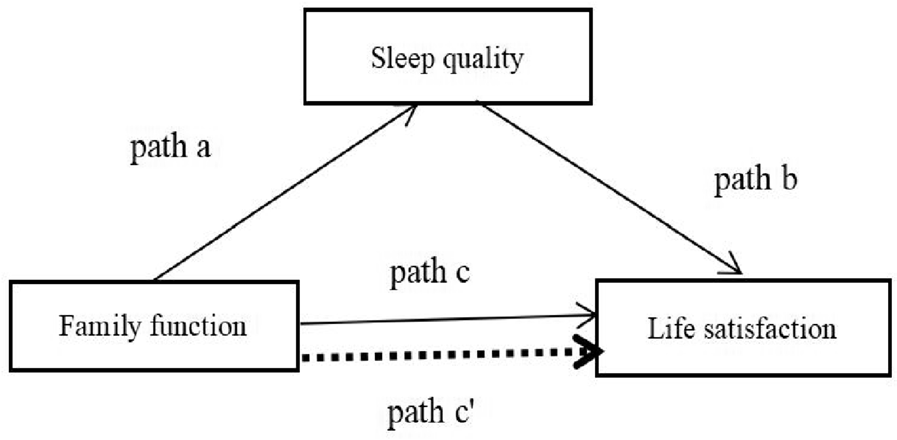
The proposed mediation model. Path a, effect of family function on sleep disturbance;path b, effect of sleep disturbance on life satisfaction path e' direct effect of family function on life satisfaction when sleep distance as a mediator;path c,total effect of family function on life satisfaction

Data analyses were performed using SPSS 26.0. Continuous data were calculated with the use of means with standard deviations (SD) and frequencies and percentages for categorical data. Bivariate Pearson’s correlation analysis was used to analyze the correlations among variables. Stepwise multiple linear regression was used to explore the effects of family function and sleep quality on life satisfaction. Social demographic variables were entered into the regression model to control the effects of covariates. The bootstrapping procedure in the SPSS PROCESS macro version 4.0 was used to examine the mediating effect of sleep quality on the relationship between family function and life satisfaction by regression-based mediation analysis. For mediation analysis, the bootstrap method can provide much greater statistical power [48, 49]. Figure 1 shows the proposed mediation model and the independent variable (family function), mediator (sleep quality), and dependent variable (life satisfaction) (Fig. 1). In this model, a is the regression coefficient predicting the effect of family function on sleep quality, and b is the regression coefficient predicting the effect of sleep quality on life satisfaction. Coefficient c is the regression coefficient predicting the total effect of family function on life satisfaction. The total effect (c) comprises a direct effect (c') of family function on life satisfaction and an indirect effect (a and b) of family function on life satisfaction through sleep quality. The estimate of direct, indirect and total effects was based on 5000 bootstrapping resamples, and a 95% bias-corrected confidence interval (CI) was constructed. If the 95% CI did not contain zero, the effect was considered significant. *P* value <0.05 was considered to indicate statistical significance.

## Results

### Characteristics of participants

Among the 140 eligible participants agreed to participate, 13 participants did not complete the questionnaires for personal reasons. Thus, the response rate was 90.7% (127/140). The mean age was 81.54 (SD=7.94) years, with a range from 60 to 93 years. Seventy-four percent of the participants were female, 57.5% of the elderly were married (with a surviving spouse), and the proportion of the education level (lower than high school) was 57.5%. The characteristics of the participants are shown in Table 1. As shown in Table 1, the mean APGAR, LSI-A and PSQI scores were 5.58 (SD=1.73), 12.54 (SD=3.57) and 7.58 (SD=3.77), respectively, suggesting that the elderly individuals in this study had low family function, low life satisfaction, and poor sleep quality.

**Table 1:** Sample characteristics (*n*=127)

Characteristics	N(%)	Mean(SD)
Age(years)		81.54 (7.935)
60-69	15 (11.8%)	
70-79	24 (18.9%)	
80-89	77(60.6%)	
≥90	11(8.7%)	
Gender		
male	33(26%)	
female	94(74%)	
Education		
≤primary school	31(24.4)	
Middle school	42(33.1)	
High school	41(32.3)	
≥college	13(10.2)	
Marital status		
Married with surviving spouse	73(57.5)	
Unmarried, divorced or other	54(42.5)	
monthly pension (RMB/month)		
<2000	50(39.4%)	
2000-4000	50(39.4%)	
>4000	27(21.2%)	
Life satisfaction(LSI-A)		12.54 (3.574)
Sleep quality(PSQI)		7.58 (3.772)
Family function(APGAR)		5.58 (1.725)

### Correlations analysis

The correlation analysis showed that life satisfaction was positively correlated with family function (*r*=0.434, p<0.01) and negatively correlated with PSQI (*r* = -0.514, *p*<0.01). PSQI was found to be negatively associated with family function (*r*=-0.387, *p*<0.01). Demographic characteristic variables were not statistically significantly associated with life satisfaction, PSQI or family function. The correlation analysis are shown in Table 2.

**Table 2:** Bivariate correlations among family function, sleep quality and life satisfaction

Variables	1	2	3	4	5	6	7
1 Age	-						
2 Gender	0.193*	-					
3 Education level	-0.278**	-0.151	-				
4 Marital status	-.000	-0.087	-0.131	-			
5 Monthly pension	-0.116	0.172	0.339**	-0.056	-		
6 Family function (APGAR)	0.055	-0.007	-0.077	-0.019	0.026	-	
7Sleep quality (PSQI)	0.087	-0.023	-0.038	-0.158	-0.049	-0.387**	
8 Life satisfaction (LSI-A)	-0.002	0.128	-0.022	0.048	0.039	0.434**	-0.514**

(* *p* < 0.05,** *p* < 0.01)

### Regression analysis

A stepwise multiple linear regression model was performed to explore the influencing factors of life satisfaction among older adults in nursing homes. The regression model revealed that family function (β=0.286, *p*<0.01) and PSQI scores (β=-0.397, *p*<0.01) were significant predictors of elderly people's sleep quality in nursing homes. The correlation analysis are shown in Table 3.

**Table 3:** Regression analyses for mediation effects between life satisfaction and family functioning

	Life satisfaction									
	Analysis I					Analysis II				
	Unstandardized coefficients(β)	Standard error	Standardized coefficients(β)	t	P	Unstandardized coefficients(β)	Standard error	Standardized coefficients(β)	t	P
Age	-0.48	0.696	-0.052	-0.601	0.549	-0.073	0.644	-0.009	-0.113	0.910
Gender	0.063	0.039	0.140	1.598	0.113	0.052	0.036	0.116	1.445	0.151
Education level	0.153	0.345	0.041	0.442	0.659	0.034	0.319	0.009	0.106	0.916
Marital status	0.512	0.563	0.075	0.910	0.365	0.015	0.528	0.002	0.028	0.977
Monthly pension	-0.693	0.709	-0.370	-0.978	0.330	-0.417	0.654	-0.223	-0.638	0.525
Family function	0.929	0.169	0.448	5.487	0.000**	0.593	0.171	0.286	3.468	0.001**
PSQI	-	-				-0.376	0.079	-0.397	-4.759	0.000**
R	0.469					0.588				
R2	0.220					0.346				
F	4.806**					7.801**				

(* *p* < 0.05,** *p* < 0.01)

### Mediation analysis

A bootstrap test was employed to test the hypothesized mediating effect of PSQI with 5000 random samplings. The findings about the mediation effects of PSQI between family function and life satisfaction are presented in Table 4. Family function had a significant effect on PSQI (path a: β=-0.8459, 95% CI=-1.2029, -0.4889), and PSQI had a significant effect on life satisfaction (path b: β=-0.3916, 95% CI=-0.5407, -0.2425). The total effect (path c) and direct effect (path c') of family function on life satisfaction were significant (β=0.8931, 95% CI=0.5626, 1.2235 and β=0.5618, 95% CI=0.2358, 0.8879, respectively). The coefficient for the indirect effect of family function on life satisfaction through PSQI was statistically significant (β=0.3312, 95% CI=0.1628, 0.5588). These results suggested that PSQI played a partial mediating role between family function and life satisfaction, and PSQI mediated 32.58% of the total effect of family function on life satisfaction.

**Table 4:** Coefficients and Confidence Intervals for Mediation Analysis

Independent variable	Mediator	Path a β(95% CI)	Path b β(95% CI)	Direct effect(path c') β(95% CI)	Indirect effect(path a×b) β(95% CI)	Total effect(path c) β(95% CI)
Family function Life satisfaction	PSQI	-0.8459 (-1.2029, -0.4889)**	-0.3916 (-0.5407, -0.2425)**	0.5618 (0.2358,0.8879)**	0.3312 (0.1628,0.5588)**	0.8931 (0.5626,1.2235)**

Mediation analysis with 5000 bootstrapping resamples. Models adjusted for age, gender, education level, marital status and monthly pension. (* *p* < 0.05,** *p* < 0.01)

## Discussion

This study provided evidence on the level of family function, sleep quality and life satisfaction and the relationship between these variables among older adults in NHs in China. This study offered an understanding of the measures that may potentially improve their life satisfaction. Family function and sleep quality were found to be positively associated with life satisfaction among elderly people in NHs. In addition, sleep quality partially mediated the relationship between family function and life satisfaction. The assumption among study variables was supported.

In this study, the mean LSI-A score was approximately 62% of the total score. Similar to previous studies [50, 51] on life satisfaction of older adults in NHs, the score was lower than that of elderly people in the community [10], indicating poor life satisfaction among this population.Trybusińska et al.'s study also showed that older adults in NHs had a lower level of life satisfaction [52]. Many factors may contribute to the decreased life satisfaction of elderly individuals, such as health status, changes in living environment, emotional satisfaction, and family relationships [11, 12], quality of service provided by nursing home staff [53]. Our findings suggest that measures should be considered to improve the life satisfaction of older adults in NHs.

As shown in the hypothesis, family function was considered an important factor affecting elderly people' life satisfaction in NHs. This result supported the previous studies that family function had positively association with life satisfaction in older adults [14, 54]. Family function has been identified as a key factor in achieving better life satisfaction. First, family function may enhance individuals’ perception of well-being and mental health and lead to better life satisfaction [55]. Second, family function has been identified to improve health promotion behavior and health conditions and further promote life satisfaction [11, 56].

In this study, the mean APGAR score was 5.58 (SD=1.725),consistent with previous research on family function of older adults in NHs [57], which was lower than that of elderly individuals living at home [11, 54]. This can be explained by the fact that on the one hand, elderly individuals in NHs leave the familiar living environment and family, and their emotional and care needs increase [58, 59]. On the other hand, children of elderly adults were busy with work and visited them less frequently [58]. Therefore, older adults in NHs reported lower APGAR score.With the lack of staff in NHs, as well as their lack of knowledge and cultural level and other reasons in NHs in China, the service provided by NHs cannot meet elderly people's care and emotional needs [59, 60]. Therefore, it is important to enhance their perceived family function and emotional needs from families, friends, and staff of NHs ,ultimately to help them assimilate into a new environment and increase their life satisfaction.

Elderly people in NHs reported higher mean PSQI scores than those in the community [27, 28], indicating have poor sleep quality. In our study, poor sleep quality was negatively associated with life satisfaction, in line with previous studies [61], Sleep was a crucial restorative behavior that affects individual health and subjective well-being [62]. Sleep quality was closely related to some major psychological behaviors, including emotion regulation and interpersonal communication [63, 64]. Elderly people in NHs with better sleep quality have good health status, subjective well-being, emotion regulation ability and interpersonal communication ability and accordingly maintain better life satisfaction. Moreover, this study found that sleep quality partially mediated the effects of family function on life satisfaction, which further proved the importance of sleep quality on life satisfaction.Poor family and social support for older people in NHs might increase the risk of sleep disorders [65], good family function could maintain family relationships [66] and improve the sense of belonging in NHs [67], provided life support and created a comfortable internal and external environment for better sleep quality [68, 69]. More specifically, higher family functioning could help elderly people actively adapt to the stress caused by changes in the living environment [70], which, in turn, improved their life satisfaction.

The results of previous studies also provided evidence that sleep quality played a mediating role in elderly people's life satisfaction. Sleep quality was found to partially mediate the association between social support and life satisfaction among migrant older adults [71]. Another study supported the mediating effect of sleep quality between self-assessed living standards and mental health among older people in rural China [72]. This study provided additional evidence for the mediating role of sleep quality in life satisfaction among older adults in NHs. Sleep quality could be regulated and improved [73]. A systematic review suggested that interventions were effective in improving sleep quality, such as music interventions [74], social activity interventions [75], behavioral and exercise interventions [76]. Strategies that improve sleep quality may help promote life satisfaction for the elderly in nursing homes. This study shows that demographic characteristics such as age and marital status have no effect on elderly people's life satisfaction, which is inconsistent with the results of previous studies [77, 78]. This finding is consistent with Van Damme-Ostapowicz's findings that age, education level, and gender did not affect older people's life satisfaction [5]. The possible reasons for this may be related to the different research subjects, different scales, and different sample sizes.

## Implications

Elderly people's life satisfaction has become a public concern worldwide. The findings in our study have some theoretical and practical implications for promoting life satisfaction. This study extends the mediating role of sleep quality between family function and life satisfaction. This shows that family function and sleep quality are important factors in improving life satisfaction for elderly people in NHs. The mediation model implies that the integrated intervention of sleep quality and family function may be more effective in promoting life satisfaction. The suggested measures are as follows: First, family members should be encouraged to visit the elderly frequently and to care about their physical and psychological status to improve their perception of family function.Second, more interest group activities can be organized in NHs to enhance their communication and sense of belonging, such as Taiji exercise, singing and drawing. This may compensate for the lack of family function and improve sleep quality. Third, it is essential to emphasize carrying out sleep intervention training to improve sleep quality for elderly individuals, such as correct cognition of sleep, regular work and rest time, and creating a comfortable sleep environment.

### Limitations

This study had some limitations. First, participants were selected in one city and a convenience sampling

method was employed in this study; thus, these results may have bias and limit generalizability. Second, this

was a cross-sectional study, the study results could preclude causal interpretations among the variables, so

a longitudinal design can be considered for future studies to examine the detailed causal relationship. Third, this

study used the form of a self-report questionnaire, which may lead to reporting bias.Fourth, we did not select older adults who had just entered nursing homes as study subjects. This may have resulted in interventions that are not appropriate for this population. In the next phase of the study, we will include nursing home residents at different stages of adaptation (within 3 months, 3-6 months, and more than 6 months) as study subjects. This approach is in line with Wang et al.'s recommendations [79], the confusing and adjustment periods for nursing home residents occur within the first three months after admission,the acceptance period falls between three to six months post-admission, and the adaptation period begins after six months of admission. We will investigate the factors that affect the sleep quality, life satisfaction, and family functioning of these three groups of participants, explore their interrelationships and compare the similarities and differences among them. More specific interventions will be proposed based on the various stages of adaptation experienced by older adults.Last,other factors may play a significant role in elderly people's life satisfaction and sleep quality,such as the disease diagnosis,activities of daily living(ADL),residence duration in NHs.In future studies, we will consider adding the effects of these factors on life satisfaction and sleep quality of older adults in NHs,in order to explore the mechanism of these factors on life satisfaction and sleep quality and to promote the improvement of the quality of sleep and life satisfaction of older adults in NHS.

## Conclusion

This study revealed that family function and sleep quality were significant predictors of elderly people's life satisfaction in NHs. Sleep quality partially mediated the relationship between family function and life satisfaction. The results of the study expanded the understanding of the interaction mechanism of family function and sleep on life satisfaction in this population. Therefore, it is recommended that interventions focused on promoting family function and improving sleep quality may be more helpful in improving elderly people's life satisfaction in NHs.

## Data Availability

Please contact corresponding author for data requests

## Abbreviations

PSQI: Pittsburgh Sleep Quality Index
LSI-A: Life Satisfaction Index A;
SD: Standard Deviation
NH: Nursing Home
